# Supplementary material for: Inherent spatiotemporal uncertainty of renewable power in China
Source: Nat Commun. 2023 Sep 4;14:5379. doi: 10.1038/s41467-023-40670-7 (PMC10477199; doi:10.1038/s41467-023-40670-7)
Supplement: Supplementary file 1 — Supplementary Information [file 41467_2023_40670_MOESM1_ESM.pdf]

# Supplementary Information

**Inherent Spatiotemporal Uncertainty of Renewable Power in China**

**Jianxiao Wang, Liudong Chen, Zhenfei Tan, Ershun Du, Nian Liu, Jing Ma, Mingyang Sun, Canbing Li, Jie Song, Xi Lu, Chin-Woo Tan, Guannan He.**

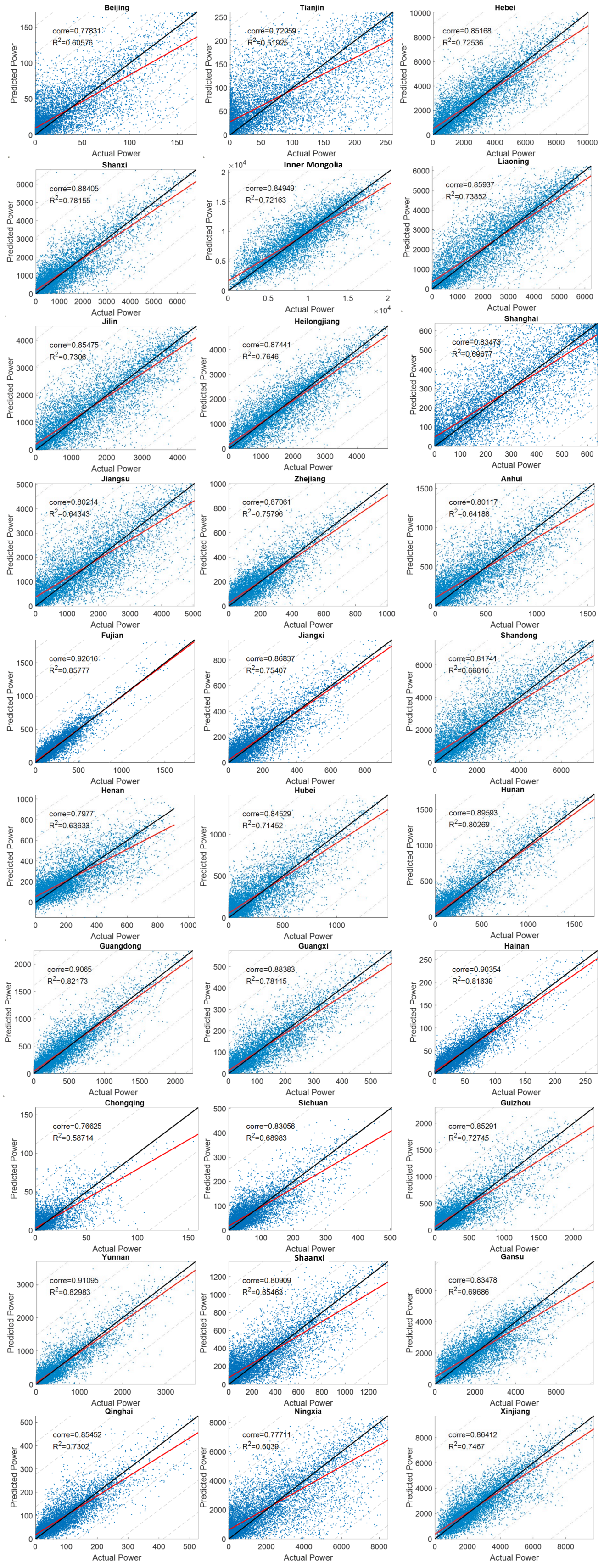

**Supplementary Figure 1.** Wind prediction error analysis of 30 provinces of China. The black solid line means diagonal, and the black dot line is the Diagonal parallel line. The red line is the fitting line of blue scatter points. Abbreviations: Corre: Pearson correlation coefficient; $R^2$: Coefficient of determination.

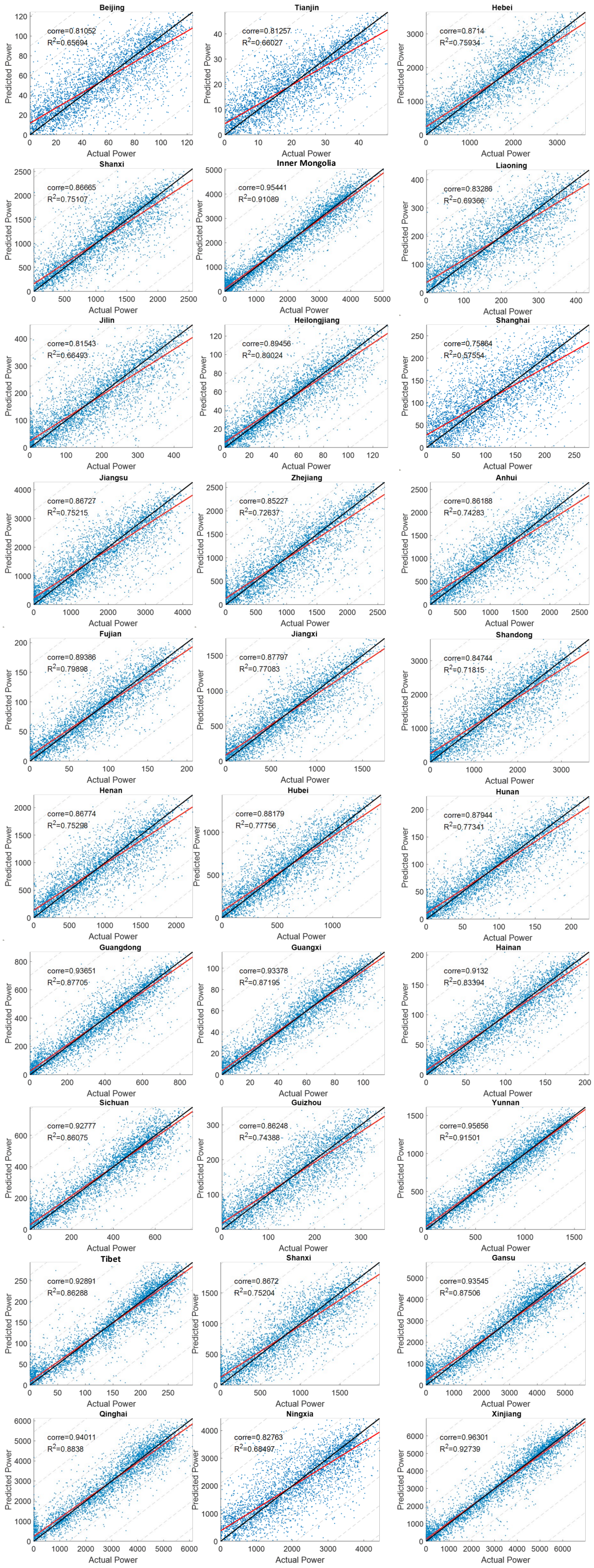

**Supplementary Figure 2.** Solar prediction error analysis of 30 provinces of China. The black solid line means diagonal, and the black dot line is the Diagonal parallel line. The red line is the fitting line of blue scatter points. Abbreviations: Corre: Pearson correlation coefficient; $R^2$: Coefficient of determination.

a

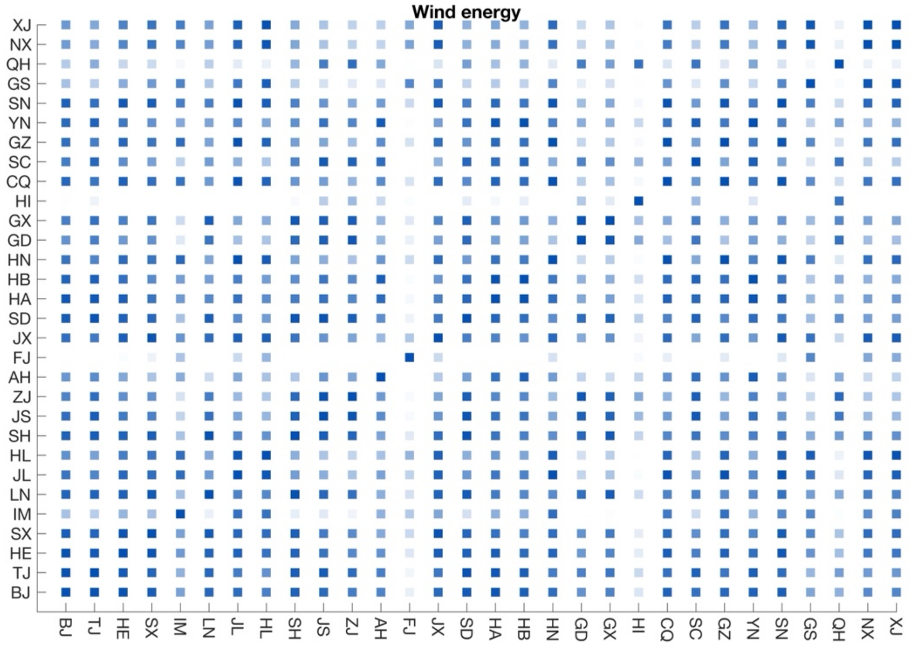


b

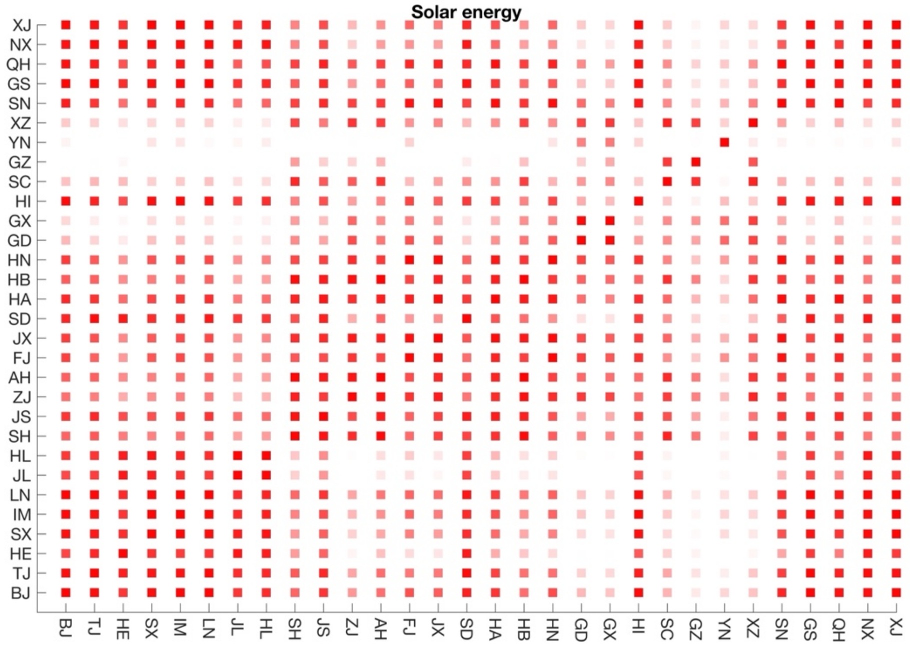


**Supplementary Figure 3. Correlation of prediction error between provinces. a,** Wind energy. **b,** Solar energy. Darker color means strong relationships between two provinces. Abbreviations: BJ: Beijing; TJ: Tianjin; HE: Hebei; SX: Shanxi; IM: Inner Mongolia; LN: Liaoning; JL: Jilin; HL: Heilongjiang; SH: Shanghai; JS: Jiangsu; ZJ: Zhejiang; AH: Anhui; FJ: Fujian; JX: Jiangxi; SD: Shandong; HA: Henan; HB: Hubei; HN: Hunan; GD: Guangdong; GX: Guangxi; HI: Hainan; CQ: Chongqing, XZ: Tibet; SC: Sichuan; GZ: Guizhou; YN: Yunnan; SN: Shaanxi; GS: Gansu; QH: Qinghai; NX: Ningxia: XJ: Xinjiang.

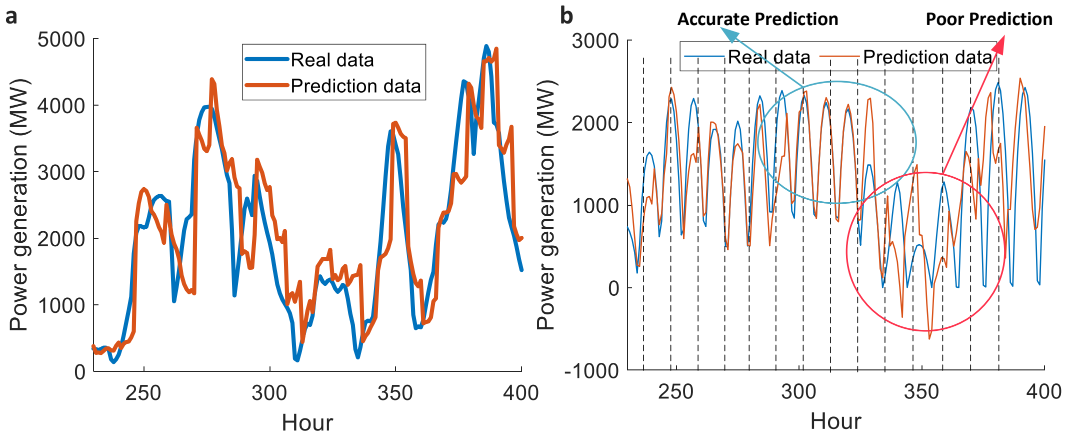


**Supplementary Figure 4. Analysis of different wind and solar power generation characteristics**. **a**, Wind power generation of Shanxi (SX) Province from 230~400 hours (total is 8760 hours) in 2016. **b**, Solar energy generation of SX from 230~400 hours. The black dot line separates each day in the time horizon.

**Supplementary Tables**

Supplementary Table 1  Mean value in Fig. 1c

| Method | Wind Prediction error (%) | Solar Prediction error (%) |
|---|---|---|
| ARIMA | 6.16 | 6.70 |
| FCNN | 6.79 | 9.53 |
| RF | 5.44 | 7.60 |
| RNN | 5.18 | 7.16 |
| SVM | 7.18 | 8.34 |

ARIMA: autoregressive integrated moving average; RF: random forest; RNN: recurrent neural network; FCNN: fully-connected neural network; SVM: support vector machine

Supplementary Table 2  Data distribution for each interval of the box diagram in Fig. 4

| Province | Interval | Max value | Min value | Average value |
|---|---|---|---|---|
| Tianjin wind | 1 | 0.329 | 0 | 0.106 |
| | 2 | 0.349 | 0 | 0.120 |
| | 3 | 0.391 | 0 | 0.132 |
| | 4 | 0.488 | 0 | 0.151 |
| | 5 | 0.539 | 0 | 0.170 |
| | 6 | 0.557 | 0 | 0.174 |
| | 7 | 0.633 | 0 | 0.189 |
| | 8 | 0.721 | 0 | 0.215 |
| | 9 | 0.664 | 0 | 0.195 |
| | 10 | 0 | 0 | 0 |
| | Total | 0.721 | 0 | 0.215 |
| Shandong wind | 1 | 0.205 | 0 | 0.068 |
| | 2 | 0.236 | 0 | 0.079 |
| | 3 | 0.273 | 0 | 0.087 |
| | 4 | 0.339 | 0 | 0.110 |
| | 5 | 0.384 | 0 | 0.115 |
| | 6 | 0.382 | 0 | 0.119 |
| | 7 | 0.404 | 0 | 0.129 |
| | 8 | 0.434 | 0 | 0.141 |
| | 9 | 0.276 | 0 | 0.107 |
| | 10 | 0 | 0 | 0 |
| | Total | 0.434 | 0 | 0.141 |
| Shanxi wind | 1 | 0.104 | 0 | 0.036 |
| | 2 | 0.169 | 0 | 0.055 |
| | 3 | 0.234 | 0 | 0.071 |
| | 4 | 0.309 | 0 | 0.095 |
| | 5 | 0.278 | 0 | 0.090 |
| | 6 | 0.288 | 0 | 0.102 |
| | 7 | 0.263 | 0 | 0.083 |
| | 8 | 0.174 | 0 | 0.052 |
| | 9 | 0.192 | 0 | 0.059 |
| | 10 | 0 | 0 | 0 |
| | Total | 0.309 | 0 | 0.102 |
| Gansu wind | 1 | 0.112 | 0 | 0.036 |
| | 2 | 0.118 | 0 | 0.037 |
| | 3 | 0.163 | 0 | 0.049 |
| | 4 | 0.188 | 0 | 0.056 |
| | 5 | 0.252 | 0 | 0.081 |
| | 6 | 0.220 | 0 | 0.076 |
| | 7 | 0.158 | 0 | 0.099 |
| | 8 | 0 | 0 | 0 |
| | 9 | 0 | 0 | 0 |
| | 10 | 0 | 0 | 0 |
| | Total | 0.252 | 0 | 0.099 |
| Beijing solar | 1 | 0.277 | 0 | 0.092 |
| | 2 | 0.259 | 0 | 0.091 |
| | 3 | 0.273 | 0 | 0.088 |
| | 4 | 0.310 | 0 | 0.098 |
| | 5 | 0.283 | 0 | 0.089 |
| | 6 | 0.273 | 0 | 0.089 |

| | | | | |
|---|---|---|---|---|
| | 7 | 0.280 | 0 | 0.095 |
| | 8 | 0.268 | 0 | 0.106 |
| | 9 | 0.644 | 0 | 0.204 |
| | 10 | 0 | 0 | 0 |
| | Total | 0.644 | 0 | 0.204 |
| Jiangsu solar | 1 | 0.170 | 0 | 0.057 |
| | 2 | 0.207 | 0 | 0.070 |
| | 3 | 0.221 | 0 | 0.071 |
| | 4 | 0.228 | 0 | 0.074 |
| | 5 | 0.216 | 0 | 0.073 |
| | 6 | 0.199 | 0 | 0.075 |
| | 7 | 0.217 | 0 | 0.083 |
| | 8 | 0.453 | 0 | 0.128 |
| | 9 | 0 | 0 | 0 |
| | 10 | 0 | 0 | 0 |
| | Total | 0.253 | 0 | 0.128 |
| Hubei solar | 1 | 0.173 | 0 | 0.057 |
| | 2 | 0.179 | 0 | 0.059 |
| | 3 | 0.206 | 0 | 0.065 |
| | 4 | 0.178 | 0 | 0.059 |
| | 5 | 0.223 | 0 | 0.067 |
| | 6 | 0.190 | 0 | 0.069 |
| | 7 | 0.197 | 0 | 0.074 |
| | 8 | 0.156 | 0 | 0.065 |
| | 9 | 0 | 0 | 0 |
| | 10 | 0 | 0 | 0 |
| | Total | 0.223 | 0 | 0.074 |
| Inner Mongolia solar | 1 | 0.104 | 0 | 0.037 |
| | 2 | 0.107 | 0 | 0.038 |
| | 3 | 0.132 | 0 | 0.044 |
| | 4 | 0.146 | 0 | 0.049 |
| | 5 | 0.143 | 0 | 0.046 |
| | 6 | 0.140 | 0 | 0.047 |
| | 7 | 0.170 | 0 | 0.053 |
| | 8 | 0.196 | 0 | 0.061 |
| | 9 | 0 | 0 | 0 |
| | 10 | 0 | 0 | 0 |
| | Total | 0.196 | 0 | 0.061 |

Supplementary Table 3 Peak ratios in each power generation interval

| Interval | Wind energy | | Solar energy | |
|---|---|---|---|---|
| | Peaks' ratio of provinces with large prediction error | Peaks' ratio of provinces with low prediction error | Peaks' ratio of provinces with large prediction error | Peaks' ratio of provinces with low prediction error |
| 1 | 0.030 | 0.005 | 0.000 | 0.000 |
| 2 | 0.030 | 0.009 | 0.004 | 0.001 |
| 3 | 0.040 | 0.017 | 0.008 | 0.006 |
| 4 | 0.048 | 0.030 | 0.022 | 0.009 |
| 5 | 0.054 | 0.044 | 0.039 | 0.026 |
| 6 | 0.056 | 0.062 | 0.063 | 0.060 |
| 7 | 0.056 | 0.012 | 0.141 | 0.141 |
| 8 | 0.058 | 0.026 | 0.278 | 0.273 |
| 9 | 0.048 | 0.111 | 0.523 | 0.370 |
| 10 | 0.031 | 0 | 0.000 | 0.000 |

**Note 1**: The provinces with large prediction error include the first three groups, i.e., prediction error larger than 5% for wind energy and larger than 6% for solar energy, and provinces with low prediction error includes the last group, i.e., prediction error lower than 5% for wind energy and lower than 6% for solar energy. The ratio is the average value of all provinces.

**Note 2**: In provinces with large prediction error, the peaks' ratio is higher in high power generation intervals, while in provinces with low prediction error, the peaks' ratio mostly concentrates on the middle power generation intervals. The phenomenon is more obvious for wind energy. The reason is that there are fewer data points for solar energy since power generation is zero for nearly half of the day, so the difference in the peaks' distribution is less significant.

Supplementary Table 4  Detailed ARIMA parameters for each province

| Province | Wind energy | | | Province | Solar energy | | |
|---|---|---|---|---|---|---|---|
| | p | q | d | | p | q | d |
| BJ | 6 | 7 | 1 | BJ | 8 | 8 | 1 |
| TJ | 6 | 6 | 1 | TJ | 8 | 9 | 1 |
| HE | 9 | 8 | 1 | HE | 9 | 7 | 1 |
| SX | 6 | 7 | 1 | SX | 9 | 8 | 1 |
| IM | 9 | 9 | 1 | IM | 9 | 8 | 1 |
| LN | 9 | 9 | 1 | LN | 9 | 9 | 1 |
| JL | 9 | 8 | 1 | JL | 9 | 8 | 1 |
| HL | 7 | 9 | 1 | HL | 9 | 9 | 1 |
| SH | 7 | 7 | 1 | SH | 7 | 8 | 1 |
| JS | 6 | 9 | 1 | JS | 9 | 8 | 1 |
| ZJ | 6 | 8 | 1 | ZJ | 9 | 6 | 1 |
| AH | 9 | 6 | 1 | AH | 9 | 9 | 1 |
| FJ | 8 | 7 | 1 | FJ | 9 | 9 | 1 |
| JX | 9 | 9 | 1 | JX | 9 | 9 | 1 |
| SD | 8 | 8 | 1 | SD | 9 | 7 | 1 |
| HA | 6 | 9 | 1 | HA | 9 | 8 | 1 |
| HB | 9 | 8 | 1 | HB | 9 | 9 | 1 |
| HN | 6 | 7 | 1 | HN | 8 | 9 | 1 |
| GD | 7 | 6 | 1 | GD | 9 | 8 | 1 |
| GX | 7 | 9 | 1 | GX | 9 | 9 | 1 |
| HI | 6 | 9 | 1 | HI | 9 | 9 | 1 |
| CQ | 9 | 6 | 1 | SC | 9 | 6 | 1 |
| SC | 9 | 6 | 1 | GZ | 9 | 8 | 1 |
| GZ | 6 | 6 | 1 | YN | 9 | 9 | 1 |
| YN | 8 | 6 | 1 | XZ | 9 | 9 | 1 |
| SN | 8 | 9 | 1 | SN | 9 | 9 | 1 |
| GS | 8 | 8 | 1 | GS | 7 | 9 | 1 |
| QH | 6 | 9 | 1 | QH | 9 | 8 | 1 |
| NX | 8 | 6 | 1 | NX | 9 | 7 | 1 |
| XJ | 8 | 6 | 1 | XJ | 9 | 9 | 1 |

ARIMA: autoregressive integrated moving average; BJ: Beijing; TJ: Tianjin; HE: Hebei; SX: Shanxi; IM: Inner Mongolia; LN: Liaoning; JL: Jilin; HL: Heilongjiang; SH: Shanghai; JS: Jiangsu; ZJ: Zhejiang; AH: Anhui; FJ: Fujian; JX: Jiangxi; SD: Shandong; HA: Henan; HB: Hubei; HN: Hunan; GD: Guangdong; GX: Guangxi; HI: Hainan; CQ: Chongqing, XZ: Tibet; SC: Sichuan; GZ: Guizhou; YN: Yunnan; SN: Shaanxi; GS: Gansu; QH: Qinghai; NX: Ningxia: XJ: Xinjiang.

Supplementary Table 5.1 Detailed parameters for random forest (RF)

| | Prediction step | Max_depth | N_estimators | Max_features |
|---|---|---|---|---|
| RF Solar | 2 hours | 1000 | 500 | sqrt |
| | 6 hours | 500 | 1000 | sqrt |
| | 24 hours | 100 | 1000 | sqrt |
| RF Wind | 2 hours | 100 | 500 | sqrt |
| | 6 hours | 500 | 500 | sqrt |
| | 24 hours | 100 | 500 | sqrt |

Supplementary Table 5.2 Detailed parameters for recurrent neural network (RNN)

| | Prediction step | Batch_size | Epochs | Learning_rate | Neurons | Activation |
|---|---|---|---|---|---|---|
| RNN Solar | 2 hours | 32 | 50 | 0.001 | 252 | relu |
| | 6 hours | 32 | 50 | 0.001 | 252 | relu |
| | 24 hours | 32 | 50 | 0.001 | 252 | relu |
| RNN Wind | 2 hours | 32 | 50 | 0.001 | 252 | relu |
| | 6 hours | 32 | 50 | 0.001 | 252 | relu |
| | 24 hours | 32 | 50 | 0.001 | 252 | relu |

Supplementary Table 5.3 Detailed parameters for fully-connected neural network (FCNN)

| | Prediction step | Batch_ size | Epochs | Learning_ rate | Neurons | Activation | Dropout_ rate |
|---|---|---|---|---|---|---|---|
| FCNN Solar | 2 hours | 64 | 50 | 0.001 | 252 | relu | 0.2 |
| | 6 hours | 32 | 50 | 0.001 | 252 | relu | 0.2 |
| | 24 hours | 64 | 50 | 0.001 | 252 | relu | 0.2 |
| FCNN Wind | 2 hours | 64 | 50 | 0.001 | 252 | relu | 0.2 |
| | 6 hours | 64 | 50 | 0.001 | 252 | relu | 0.2 |
| | 24 hours | 32 | 50 | 0.001 | 252 | relu | 0.4 |

Supplementary Table 5.4 Detailed parameters for support vector machine (SVM)

| | Prediction step | Estimator_C | Estimator_epsilon | Estimator_gamma |
|---|---|---|---|---|
| SVM Solar | 2 hours | 1000 | 0.01 | 0.01 |
| | 6 hours | 100 | 0.01 | 0.01 |
| | 24 hours | 100 | 0.01 | 0.01 |
| SVM Wind | 2 hours | 10 | 0.01 | 0.01 |
| | 6 hours | 100 | 0.01 | 0.01 |
| | 24 hours | 100 | 0.01 | 0.01 |

**Supplementary Note 1: Abbreviation of 30 provinces in China**

This study focuses on 30 provinces of China, except Chongqing solar, Tibet wind, Taiwan, Hong Kong, and Macao province, due to the data limited and unsuitable to build wind energy and solar energy, including: Beijing (BJ), Tianjin (TJ), Hebei (HE), Shanxi (SX), Inner Mongolia (IM), Liaoning (LN), Jilin (JL), Heilongjiang (HL), Shanghai (SH), Jiangsu (JS), Zhejiang (ZJ), Anhui (AH), Fujian (FJ), Jiangxi (JX), Shandong (SD), Henan (HA), Hubei (HB), Hunan (HN), Guangdong (GD), Guangxi (GX), Hainan (HI), Chongqing (CQ) wind, Tibet (XZ) solar, Sichuan (SC), Guizhou (GZ), Yunnan (YN), Shaanxi (SN), Gansu (GS), Qinghai (QH), Ningxia (NX), Xinjiang (XJ).

**Supplementary Note 2: Detailed content of each category divided by prediction error**

We divide four categories for wind and solar energy, respectively, according to the prediction error. For the wind energy, the four categories and their content are expressed as follows:

i) >9%, including TJ, SH, JS, NX;

ii) 7%~9%, including BJ, LN, JL, AH, SD, HA;

iii) 5%~7%, including HE, SX, IM, LJ, JX, HB, GX, SN;

iv) <5%, including ZJ, FJ, HN, GD, HI, CQ, SC, GZ, YN, GS, QH, XJ.

For solar energy, the four categories and their content are expressed as follow:

i) >8%, including BJ, SH, TJ, NX, LN, JL;

ii) 7%~8%, including HE, SX, JS, ZJ, SD, GZ, SN;

iii) 6%~7%, including AH, JX, HA, HN, HB;

iv) <6%, including IM, LJ, FJ, GD, GX, HI, SC, YN, XZ, GS, QH, XJ

**Supplementary Note 3: Detailed content of North, East, Central, and Southeast area of China**

To analyze the nationwide distribution of prediction error, we specify four areas of China, which is shown as follows:

i) North area of China: BJ, TJ, LN, JL, SX, HE;

ii) East area of China: SD, SH, JS, AH, HA;

iii) Central area of China: NX, SN, HB, JX, HN;

iv) Southeast area of China: CQ, SC, YN, GX, GZ.

**Supplementary Note 4: Explanation of hourly wind energy and daily solar energy**

We analyze the data feature, concluding the characteristic of wind and solar output: the wind output fluctuates greatly, and presents irregular distribution on the hourly scale, while the solar output regularly distributes on the daily scale, increasing from an average 1000 Megawatt (MW) to an average 1500MW, then drop to average 500MW in a day, as shown in Supplementary Fig. 3, after excluding the near zero output of solar energy (The reason is that the solar output is near zero at nighttime, which is unnecessary to predict and will greatly affect the prediction results). These differences are attributed to the properties of the sun and wind, with irregular wind throughout the day, while the sun rises and sets each day, and its radiation varies accordingly. Based on these characteristics, hourly prediction for solar power is more accurate than wind power most of the time. The reason for the inaccurate solar energy prediction is the

difference between each day. For example, the peak of several consecutive days (310-330 hours in Fig. 3b), with the difference within 100MW, drops to a lower peak (340-360 hours in Fig. 3b), with a difference of more than 1000MW (such as cloudy days), and the average prediction error also increases from 1.61% to 18.76%. Thus, the solar power fluctuation that affects prediction should be seen on a daily scale. In this work, we use hourly and daily power generation data to analyze wind and solar prediction errors, respectively.

**Supplementary Note 5: Parameter tunning explanation of random forest (RF), recurrent neural network (RNN), fully-connected neural network (FCNN), and support vector machine (SVM)**

We use the GridSearch approach to hyperparameter tuning to optimize the performance of machine learning models, including RF, RNN, FCNN, and SVM, for wind and solar generation prediction. Our methodology involves an exhaustive search over a range of hyperparameters to identify the optimal combination that maximizes model performance on a validation set. Cross-validation is employed to ensure an unbiased evaluation of model performance. The wind and solar generation data from 30 provinces in China are combined into two national datasets, further divided into training and testing data. For each type of prediction step (2-hour, 6-hour, and 24-hour intervals), the models are tuned separately using GridSearch. Specific hyperparameters are optimized for each model. For example, RF's hyperparameters include the number of trees, maximum tree depth, and maximum features, while SVM includes regularization parameters. RNN's hyperparameters encompassed the number of neurons, learning rate, batch size, and activation function, while FCNN's hyperparameters includes the number of neurons, learning rate, epochs, dropout rate, and batch size. Our approach results in a significant improvement in model performance compared to the default models, increasing model efficiency.
